# Acidity of rainfall samples in close vicinity of coal-fired power plant with wet cooling tower with flue gas injection

**DOI:** 10.1007/s10661-021-09443-x

**Published:** 2021-09-07

**Authors:** Katarzyna Korszun-Klak, Stanislaw Hlawiczka, Rafal Kobylecki

**Affiliations:** 1 Faculty of Infrastructure and Environment, Czestochowa University of Technology, 42-201 Czestochowa, Poland; 2 Institute for Ecology of Industrial Areas, 40-844 Katowice, Poland

**Keywords:** Acid rain, Coal-fired power plant, Cooling towers

## Abstract

The paper presents measurement data concerning the degree of acidification of precipitation collected during a 6-month measurement campaign carried out in an immediate vicinity of a power plant, where the cooling tower was used for discharging flue gases as a product of coal combustion. As reference, data obtained from parallel measurements carried out at a monitoring station considered as city background station were used. High acidity of precipitation was anticipated due to reactions of acid gases contained in the combustion gases with water, which already occur inside the cooling tower. The results have not confirmed this assumption. The pH value of the precipitation samples was significantly higher than the pH of rainwater at the background station located 18 km away from the power plant.

## Introduction

Studies on the causes of precipitation acidity have been conducted for a long time, and there is no doubt that precipitation with a pH less than 5.6 must contain acids of anthropogenic origin (Likens, 1976; Charlson & Rhode, 1982; Seinfeld, 1976). The causes of acid deposition may in particular be found due to emissions of flue gas from the combustion of solid fuels in coal power plants (Granat, 1972; Hewitt, 2001; Srivastava et al., 2004). Power plants are a dominant emission source of SO_2_, NO_*x*_, CO_2_ and HCl, i.e. chemical compounds that lead to formation of acid rain. In plumes emitted mostly from coal-fired power plants, there are favourable conditions for chemical changes leading to the formation of acid-forming compounds. The main driving factor is the accumulation of reactive compounds and substances that demonstrate catalytic properties (Neuman et al., 2004; Frost et al., 2006; Sillman, 2000; Springston et al., 2005; Hlawiczka et al., 2003, Zhou et al., 2012).

Acid oxides, especially SO_2_ and NO_2_, emitted from power plant stacks undergo chemical changes when transported with plume into the atmosphere. In the absence of clouds, the key pathway for the SO_2_ removal from power plant plumes is gas-phase SO_2_ oxidation (Springston et al., 2005). These changes may also lead to particle growth (Brock et al., 2002, 2003). When SO_2_ comes into contact with water (cloud, rain droplets), sulphur oxides convert to sulphates via aqueous reactions. Flue gas emitted by power plants may also include SO_3_ whose reaction with water results in formation of a strong sulphuric acid (Srivastava et al., 2004). The degree of acidity is naturally regulated by the presence of some neutralizing components contained in the power plant plumes that include alkaline compounds, such as calcium, magnesium, sodium, potassium compounds and ammonium compounds. They are constituents of dust that has not been caught by the dust removal systems (Brueggemann & Rolle, 1998; Flues et al., 2002; Hlawiczka et al., 2003; Hlawiczka & Korszun, 2012).

Acid rain is typically attributed with a substantial nuisance, as the acidifying compounds emitted from high power plant stacks may acidify the environment even at large distances from the emission sources (Hlawiczka et al., 1994; Schopp et al., 2003; Menz & Seip, 2004; EMEP, 2014; EEA, 2017). At the same time, an extensive review of professional literature concerning the impact of power plant emissions on the environment surprisingly indicates that it is difficult to find data about the formation of acid rain in the closest vicinity of power plants. From a physical and chemical perspective, the following two cases promote formation of a strongly acidic rain:

- Case 1: when a non-dispersed flue plume mixes with a vapour plume from a wet cooling tower. Highly concentrated flue gas constituents that have not been diluted with air yet come into contact with warm water droplets emitted from a wet cooling tower present in the plume creating ideal conditions for a rapid and effective reaction;
- Case 2: when a wet cooling tower is used to remove flue gas to the air (Ecksteinet al., 1997; Harte & Kratzig, 2002; Busch et al., 2002; Harte & Wittek, 2009). Highly dispersed water at elevated temperature reacts with acidic flue gas components creating favourable conditions for formation of acid products already inside the cooling tower, that is, even before the plume leaves it.

A strongly acidic precipitation in a vicinity of a power plant causing an adverse impact on the environment and surface of anthropogenic structures may be amplified by a rainfall occurring simultaneously with one of the cases as presented above. The rainfall brings then the acid reaction products present in the plume emitted from a wet cooling tower that discharges flue gases (case 2) or in mixed plumes from a cooling tower and a power plant stack (case 1) to the earth’s surface resulting in strongly acidic depositions. The validity of the thesis has already been confirmed for case 1 by some measurement data (Hlawiczka et al., 2016). The lowest pH value observed in these studies was on the level of 1.4 which proved that such drastic episodes may take place.

Referring to case 2, the possible formation of concentrated acid vapour where a natural-draft wet cooling tower is used for discharging flue gases is less evident, being subject for consideration in this study. So far, the topic has been examined solely from the perspective of its impact on the construction of a cooling tower with a main focus on the corrosion aspect of the inner surface of the shell which can be attracted by acid vapour (Bush, 2001; Harte et al., 2005; Harte & Wittek, 2009; Chen et al., 2017). These studies refer to the issues such as thermal stresses of the shell’s cross section, less ductility against dynamic wind action and thus additional cracking problems and how well flue gases are mixed with the plume (Eldredge et al., 2015; Jahangiri & Golneshan, 2001; Busch et al., 2002). It was also found that under strong wind conditions, the centrally introduced flue gas can flow close to the tower shell due to inflow of cold air and bending of the gas (air and flue gas) stream. All these factors may increase the corrosion risk of the tower shell and indicate that the application of a corrosion-resistant liner is required (Klimanek et al., 2015). These studies, however, do not provide any measurement data that would illustrate the acidity level of a plume leaving a wet cooling tower used for discharging flue gases from coal combustion, as considered in case 2. This study presents such data collected from a half-year measurement campaign carried out in a close vicinity of a coal-fired power plant where a wet cooling tower is used to remove flue gas to the air.

## Material and methods

### Sampling sites

Precipitation samples were collected for a period of 6 months (from June 2013 to December 2013) at two measuring stations: in Jaworzno (17 samples) and Katowice (17 samples). The measurement station in Jaworzno was located at the premises of a coal-fired power plant, in an immediate vicinity of the cooling towers which were used for discharging flue gases. The station in Katowice, considered as a background station, served as a reference for the results obtained in Jaworzno. Measurements in Katowice were carried out at an air quality monitoring station operated by the Institute for Ecology of Industrial Areas (IETU). The station is located at the Institute’s premises, 5 km westward from the centre of the Katowice city, 18 km northwest from power plant in Jaworzno, southern Poland (50.258 N, 19.8 E).

Fuel combustion plant PKE S.A. Elektrownia Jaworzno III consists of six pulverized fuel boilers OP-650 with a total capacity of 1345 MW_e_ fired with a mixture of coal and biomass, the fraction of which does not exceed 10%. Mixed coal and biomass are milled in a mill unit then dried and fed into the boiler, including air. The boiler is equipped with 24 burners arranged in four rows in the front wall. In addition, each boiler has 12 oil burners used to fire it up.

There are 3 electrostatic precipitators installed in each boiler with a total dust removal efficiency of 99–99.7%. Three centrifugal flue gas fans are installed behind them. Flue gas from boilers is cleaned in a desulphurization installation using limestone in three independent processes. This installation reduces SO_2_ emissions by over 95%. Sulphur removal consists in absorption of sulphur dioxide in an aqueous limestone suspension. A dry solid product is obtained—synthetic gypsum—as a result of the general chemical equation:

CaCO_3_ + SO_2_ + 1/2 O_2_ + 2 H_2_O → CaSO_4_ × 2 H_2_O ↓ + CO_2_↑

Flue gas after dust removal in a three-zone electrostatic precipitator system and cleaning in a desulphurization installation is discharged into the air by three cooling towers, h = 120 m and diameter d = 54.7 m each. This solution was introduced in 1996 for four out of six blocks, whereas in the case of the two remaining blocks flue gas has been discharged through cooling towers since 2008.

Water in the power plant cooling system is used as process water. Its parameters are shown in Table 1.

**Table 1:** Parameters of water in the power plant cooling system (data: Jaworzno III Power Plant)

Parameter	Unit	Min	Max	Average
General alkalinity	mval/l	1.10	2.30	1.35
Carbonate hardness	mval/l	1.10	1.65	1.35
Non-carbonate hardness	mval/l	7.55	13.15	9.50
Total hardness	mval/l	8.65	14.65	10.85
Calcium hardness	mval/l	4.25	8.95	6.00
pH	-	7.9	8.3	8.05
Specific electrolytic conductivity	μS/cm	919	1425	1091
Iron content	mg/l	0.05	0.51	0.133
COD	mg O_2_/l	5.2	17.5	9.1
Chloride content	mg/l	59.0	100.6	78.6
Sulphate content	mg/l	353	592	460
Silica content	mg/l	9.76	16.92	13.25
Ammonia content	mg/l	0.017	0.62	0.09
Total suspension content	mg/l	1.2	13.2	6.0

### Sampling and analysis

**Fig. 1:**
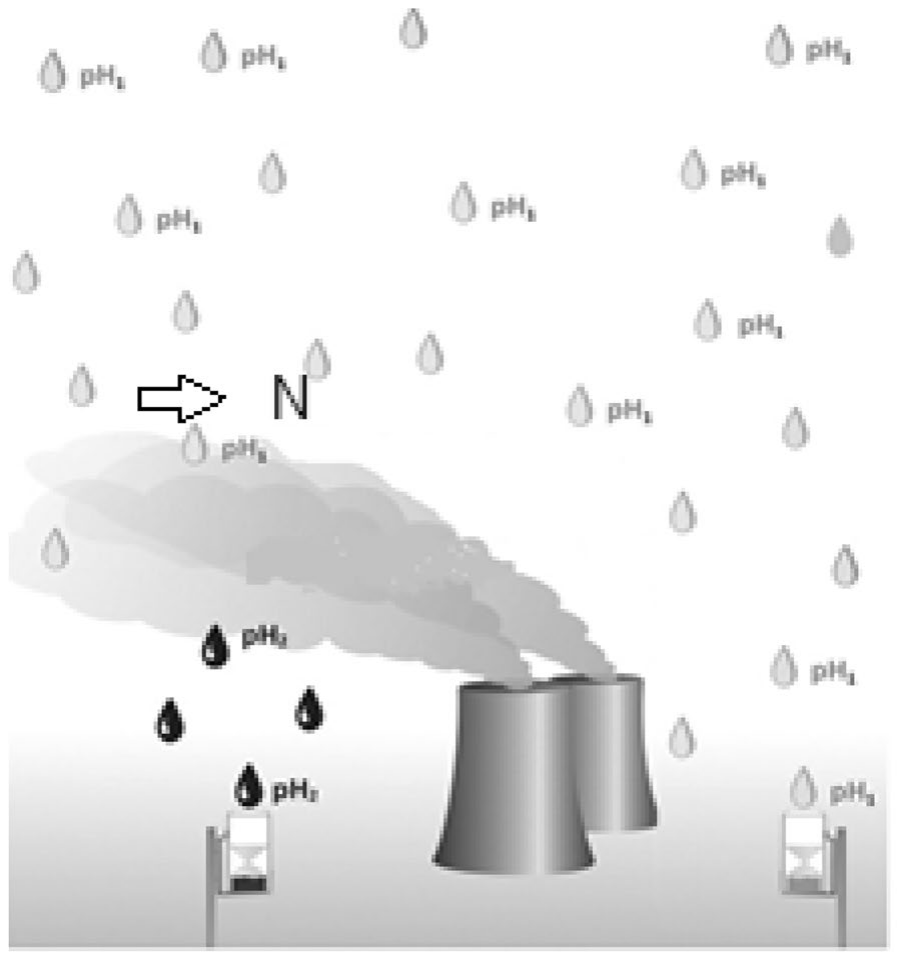
Schematic diagram of the acidity measurements of precipitation

Precipitation samples were collected in a funnel, which were located at a distance of 150 m from the cooling towers of the power plant (Fig. 1) and at the premises of the Institute for Ecology of Industrial Areas in Katowice. Daily rainfall samples were collected in bulk collectors: 5-L polyethylene bottles with 360-mm-diameter polyethylene funnels, pre-washed with high-quality deionized water. After each collection of the rainfall, its volume was measured and pH was determined using the CX-731 pH meter with an automatic temperature compensation before its filtration through 0.45-μm cellulose membrane filters. The funnel was placed on a tripod stand 1.5 m above the ground to avoid contamination of samples by soil particles during heavy rain. The samples were collected as total of dry and wet deposition. A mast with an ultrasonic anemometer at the top was located a few meters from the funnel, at a height of approx. 5 m. The anemometer was connected to a computer, which was used to collect the wind direction data.

The pH_1_ symbol in Fig. 1 refers to the background pH value of the atmospheric precipitation. The pH_2_ symbol is the pH value of the atmospheric precipitation in a close proximity to the cooling towers.

Mg^2+^, Ca^2+^, Na^+^ and K^+^ cations were determined using inductively coupled plasma optical emission spectroscopy (ICP-OES). NH_4_^+^ cation was determined using the spectrophotometric method according to PN-ISO 7150–1:2002. All analysed anions were analyzed by ion chromatography.

### Statistical analysis

A chemometric analysis of the data from the analyses of the precipitation samples collected in the vicinity of the Jaworzno power plant was conducted in order to determine the potential relationship between the wind direction and the deposition of ions determined in precipitation (Na^+^, K^+^, Mg^2+^, Ca^2+^, SO_4_^2−^, NO_3_^−^, Cl^−^, NH_4_^+^). A one-way analysis of variance (ANOVA) was applied for this purpose, using STATISTICA 13.3. Inorganic ion deposition data following logarithmic transformation of x′ = log(x), and pH values were used as a set of input data. The goal of such transformation was to remove strong asymmetry in the distribution of variables and obtain new variables with a distribution close to a normal distribution.

## Results and discussion

### pH of collected precipitation samples

The pH values of the precipitation samples from the Jaworzno sampling site (S1) ranged from 4.57 to 7.78, and those collected in Katowice (S2) varied from 4.84 to 6.81. In the case of Jaworzno, a high percentage, i.e. 47%, of the precipitation samples amounted to pH > 6.6, which is referred to as an elevated pH according to a classification suggested by authors of this article. No samples with such pH values were recorded in Katowice. Also, there were no precipitation samples at both stations that were classified as strongly acidic. Table 2 contains data on the percentage share of precipitation samples in relevant acidity categories.

**Table 2:** Range of pH value (%) in the two sampling sites: S1 (Jaworzno) and S2 (Katowice)

Categories of pH value	**S1**	**S2**
Increased pH > 6.6	Heavily acid pH < 4.4	0
Slightly increased pH 6.1–6.6	11.8	17.6
Normal pH 5.6–6.0	6	23.5
Slightly acid pH 5.0–5.5	29.4	47
Acid pH 4.4–4.9	5.8	12
Heavily acid pH < 4.4	0	0

**Fig. 2:**
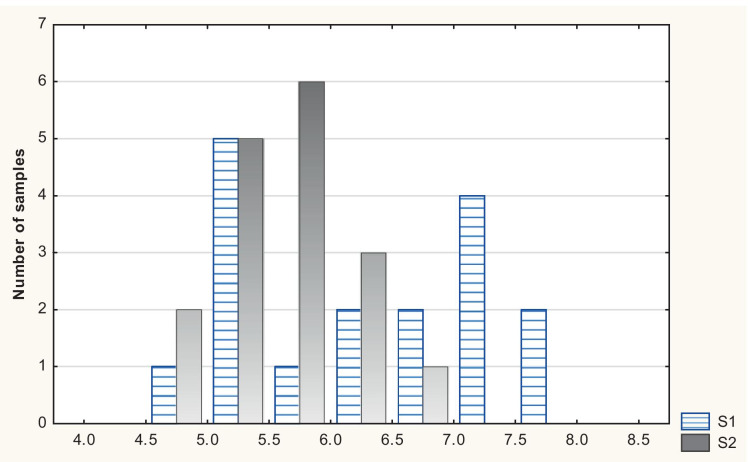
pH values of daily precipitation samples collected within 6 months of measurements in Jaworzno (S1) and Katowice (S2)

The lowest pH = 4.57 at S1 was recorded on 5 December, when the flow of air mass came from SW, i.e. a direction opposite to the cooling towers in relation to the measurement station. The precipitation pH ranging from 4.4 to 6 was prevailing at the measuring station in Katowice. At the same time, the pH values recorded for almost half of the samples collected in Jaworzno were over 6.6. A graphic layout of daily pH values of precipitation determined during the measurement campaign in Jaworzno and Katowice is presented in Fig. 2.

A comparison of the pH values of precipitation samples collected in Jaworzno and Katowice (Fig. 2) clearly indicates that the values determined for the Jaworzno site were higher. Only 6 out of the total 17 samples showed pH below 5.6 while the remaining samples demonstrated surprisingly high pH values with the highest pH reaching 7.78. Such high observed values may be even more striking especially when compared to the pH values that have been measured in precipitation occurring in the vicinity of coal-fired power plants or at industrial sites. As an example, a measurement campaign can be used which was carried out a close proximity of a coal-fired power plant where flue gases were discharged through a stack (Hlawiczka et al., 2016). The pH values of the collected precipitation samples were even below pH = 2. Other similar examples can be found for measurements carried out in a vicinity of a coal-fired power plant in Brazil, where the average yearly pH value of the precipitation was 4,7 (Flues et al., 2002).

### Wind direction versus deposition of ions

**Fig. 3:**
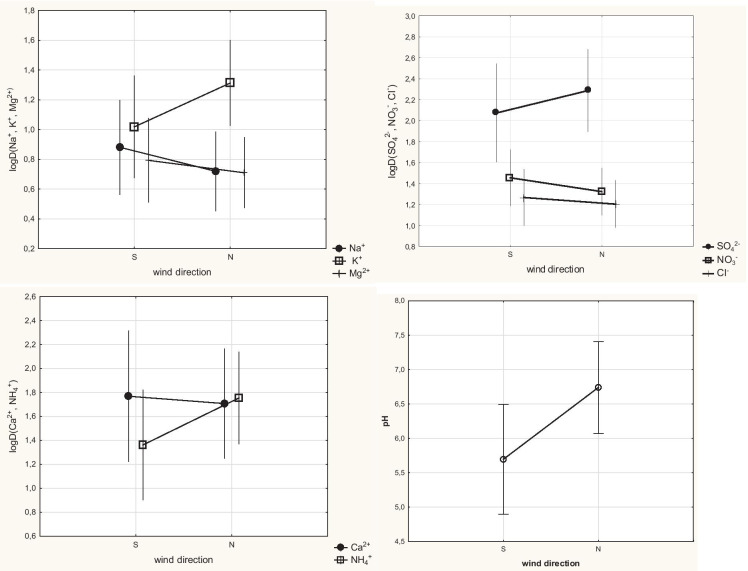
Changes of ion deposition logarithms and pH versus wind direction categories

In the conducted ANOVA analysis, only 2 distinguished categories based on the distribution of wind directions were considered from the north and from the south, although air masses also came from the south-west and north-east. It was due to the fact that the most significant part of the analysis was to identify the air flow from the direction of the power plant (i.e. from the north and the north-east which means air masses inflow above the funnel) or from the opposite direction—see Fig. 1. The analysis showed that the division into 2 classes of prevailing wind directions, with significance level α = 0.05, was not significant for the changes in the log-values of Cl^−^ deposition (see Fig. 3). Such division, however, turned out to be significant for changes in the logarithms of other ions and the pH value of precipitation samples. Significantly higher amounts of SO_4_^2−^, K^+^ and NH_4_^+^ and precipitation with higher pH came from the direction of the power plant (N). Potassium and ammonium ions present in the precipitation are highly important, because they have the greatest potential for neutralizing acidity, as confirmed by the analyses published in other articles (Hlawiczka & Korszun, 2012). This fact is also confirmed by analysing the data on the correlations between the analysed ions and pH presented in Fig. 3 and Table 3.

**Table 3:** Relationships between non-organic ions in precipitation versus pH

Variable	pH	Na^+^	K^+^	Mg^2+^	Ca^2+^	SO_4_^2−^	NO_3_^−^	Cl^−^	NH_4_^+^
pH	1.00								
Na^+^	0.09	1.00							
K^+^	0.67	0.46	1.00						
Mg^2+^	0.30	0.84	0.59	1.00					
Ca^2+^	−0.38	−0.28	−0.28	−0.33	1.00				
SO_4_^2−^	−0.33	−0.25	−0.18	−0.28	0.99	1.00			
NO_3_^−^	−0.38	0.66	−0.06	0.62	−0.19	−0.19	1.00		
Cl^−^	−0.13	0.81	0.33	0.74	−0.24	−0.20	0.85	1.00	
NH_4_^+^	0.62	0.39	0.84	0.50	−0.33	−0.24	0.05	0.38	1.00

The results presented in Table 3 made it possible to assess the power of relationships between the analytes co-occurring in the precipitation collected in the funnel placed 150 m from the cooling towers of the power plant and the pH of the samples. A positive correlation between pH and the concentrations of potassium and ammonium ions indicates that these cations cause an increase of the pH value. In turn, the positive correlation between Na^+^, Cl^−^ and NO^−^_3_ concentrations may indicate that these ions are deposited together with precipitation as NaCl and NaNO_3_. Such positive correlations between ions suggest that they should be treated as one group that originates from flue gases emitted from the coal power plant. A very strong correlation between the concentrations of sulphate and calcium ions proves that these ions react with each other and reach the ground with precipitation as CaSO_4_. At the same time, positive relationships between Mg^2+^, NO_3_^−^ and Cl − suggest that these ions may be deposited and collected in the funnel in form of MgCl_2_ and Mg(NO_3_)_2_.

## Conclusions

The basic assumption of the study was to indicate potential acidity of precipitation in the immediate vicinity of a coal power plant, in which cooling towers also serve as a stack for combustion gases. High acidity was anticipated due to reactions of acid gases contained in combustion gases with water, which already may occur inside the cooling tower. The results have not confirmed this assumption. The pH values of precipitation samples collected 150 m from the cooling towers of the power plant were significantly higher than the pH of rainwater that came from city background station 18 km away from the power plant (Table 2).

It is difficult to clearly establish the reason for these results. However, three options may be considered to justify the findings:

- Acidic products formed inside the cooling tower as a result of fuel gases mixed with the cooling water droplets in most cases fall to the bottom of the tower with only a small part reaching the plume emitted from the cooling tower into the air;
- the reaction of the cooling water may have an acidity-neutralizing effect due to its slightly alkaline character observed in the investigated power plant (Table 1). This issue still requires investigation;
- the samples collected in the funnel consisted of products formed inside the cooling tower mixed with rain drops, however, in such proportion that the resultant acidity of this mixture was closer to the acidity of the rain rather than the acidity of the plume emitted from the cooling tower.

At this stage, authors are also not aware of the results of other studies determining acidity of plumes with flue gases emitted from cooling towers with flue gas injection, during which rainfall samples would be collected very close from the emission points of these plumes. Similar investigations using methods identical to the one presented in this study were carried out for a coal-fired power plant in which combustion gases were discharged to the atmosphere though a stack instead of a cooling tower (Hlawiczka et al., 2016). A common feature for the research presented in this paper and the mentioned study is the use of rainfall as a medium transferring constituents present in the plumes to the funnel. Obviously, rainfall droplets had their own acidity which was not related to the pollution emitted from the power plant, apart from acidity brought by mixed plumes. However, the methodology applied in the studies in question made it possible to distinguish the rainfall samples not affected by the power plant emissions. The ultrasonic anemometer used in the studies and the wind field recording allowed the authors to classify rainfall samples into two types:

1. samples collected on days when the wind transferred the plume from the cooling tower towards the funnel, which at the time gathered rain droplets mixed with the constituents present in the plume; and
2. samples collected on days when wind transferred the plume away from the funnel, which at the time gathered rainfall only.

From the perspective of the purpose of this study, the ideal conditions for obtaining the results would be when the rainfall occurred at stable wind direction conditions and the rainfall containing plume with mixed flue gases and vapour from the cooling water was gathered in the funnel.

Based on the study results, it would be premature to conclude that the discharge of flue gases through cooling towers is more favourable in terms of formation of acid rain than their discharge in a traditional manner, i.e. through a stack. If it was the case, however, this would be an important argument speaking in favour of using such solutions in large industrial and power plants. So far, it has been believed that benefits resulting from the use of wet cooling towers with flue gas injection have a cost-effective and aesthetic aspect. Cost-effectiveness consists in avoiding additional energy and investment outlays resulting from the need to re-heat flue gas to the temperature above the dew point, which is necessary to prevent increased corrosion of stacks and increase the thermodynamic plume uplift. This need is a consequence of increasing the humidity and reducing the temperature of gases emitted as a result of flue gas wet desulphurization technology. If flue gas is directed to the cooling towers, huge quantities of heat could be used to transport it into higher atmosphere layers, which has a positive effect for the dispersion of the emitted pollutants.

## Data Availability

The datasets generated during and/or analysed during the current study are available from the corresponding author on reasonable request.
